# A Bayesian Approach to Genome/Linguistic Relationships in Native South Americans

**DOI:** 10.1371/journal.pone.0064099

**Published:** 2013-05-16

**Authors:** Carlos Eduardo Guerra Amorim, Rafael Bisso-Machado, Virginia Ramallo, Maria Cátira Bortolini, Sandro Luis Bonatto, Francisco Mauro Salzano, Tábita Hünemeier

**Affiliations:** 1 Departamento de Genética, Instituto de Biociências, Universidade Federal do Rio Grande do Sul, Porto Alegre, Rio Grande do Sul, Brazil; 2 Faculdade de Biociências, Pontifícia Universidade Católica do Rio Grande do Sul, Porto Alegre, Rio Grande do Sul, Brazil; George Washington University, United States of America

## Abstract

The relationship between the evolution of genes and languages has been studied for over three decades. These studies rely on the assumption that languages, as many other cultural traits, evolve in a gene-like manner, accumulating heritable diversity through time and being subjected to evolutionary mechanisms of change. In the present work we used genetic data to evaluate South American linguistic classifications. We compared discordant models of language classifications to the current Native American genome-wide variation using realistic demographic models analyzed under an Approximate Bayesian Computation (ABC) framework. Data on 381 STRs spread along the autosomes were gathered from the literature for populations representing the five main South Amerindian linguistic groups: Andean, Arawakan, Chibchan-Paezan, Macro-Jê, and Tupí. The results indicated a higher posterior probability for the classification proposed by J.H. Greenberg in 1987, although L. Campbell's 1997 classification cannot be ruled out. Based on Greenberg's classification, it was possible to date the time of Tupí-Arawakan divergence (2.8 kya), and the time of emergence of the structure between present day major language groups in South America (3.1 kya).

## Introduction

The patterns of genetic and linguistic variation have been compared for over three decades. These studies rely on the hypothesis that languages, as many other cultural traits, evolve in a gene-like manner, accumulating diversity through time and being subjected to evolutionary mechanisms of change [1], [2]. However, it should be mentioned that language, as a culturally mediated trait, is also transmitted horizontally (between unrelated individuals) in a Lamarckian way. This fact may lead to its undergoing a faster mutation rate and being subject to additional evolutionary forces [1], [3]–[5]. Thus, linguistic and genetic evolution may or may not agree [1], [6]–[13].

Studies involving Native American language and gene parallel evolutions are scarce ([3], [8], [9], [12], [14], [15] and references therein), but have brought relevant contributions to our understanding of the peopling of the Americas. However, some important parameters, such as population size differences, demographic fluctuations, or gene flow among demes, were not considered [8], [12], [15], [16].

In the present work, we revisited the problem considered by Salzano et al. [3] –*i.e.* use of genetic data to evaluate different native language classifications in South America – comparing discordant models with the current patterns of genetic variation. We propose realistic evolutionary models based on the Coalescent [17] and developed under a robust statistical framework, the Approximate Bayesian Computation (ABC; [18], [19]). Differently from earlier studies, this approach considers variances in population effective size through time, among demes, and gene flow; dates fission events, and can handle a large set of genetic markers (in the present case, 381 microsatellite loci).

In this analysis, we addressed three main questions: (a) Which language classification better fits the current South American genome-wide diversity? (b) How old are the interpopulation branch connections? and (c) Do the divergence dates between language groups, as estimated by genetic and linguistic data, agree?

## Subjects and Methods

### Linguistic classifications

From the six classifications that cover South Native American languages: Loukotka [20], Rodrigues [21], Greenberg [22], Campbell [23], Urban [24], and Lewis [25]; only three could be used here, since Rodrigues' and Urban's classification are restricted to certain groups and Lewis' to recent branches (which are identical among these classifications). Five major South American linguistic groups were considered: Andean, Arawakan, Chibchan-Paezan, Macro-Jê, and Tupí.

Loukotka [20], Greenberg [22], and Campbell [23] recognize roughly the same large language groups:

1. Andean: distributed along the Andean Cordillera (mainly Chile, Peru, and Bolivia). Examples: Aymara and Quechua;
2. Arawakan: distributed along most of the equatorial latitude. Includes the Piapoco and Wayuu;
3. Chibchan-Paezan: occupying the extreme northwestern territories of the subcontinent. Examples: Arhuaco, Kogi, and Waunana;
4. Macro-Jê: found in Central and Eastern Brazil (example, Kaingang); and
5. Tupí: distributed from the Amazon Forest southwards. Guarani is its most southern group.

Despite this agreement, each of these linguists employed different methods to classify the relationships between these groups. Greenberg [22] used multilateral comparisons, examining many languages simultaneously to detect similarities in a small number of basic words and grammatical elements. Campbell [23] used a more orthodox analysis: the comparative method, considering that proposals of remote linguistic relationships are only plausible when a series of other possible explanations have been eliminated. And finally, Loukotka [20] made use of two different methods in his classification: the lexicostatistical in some and the comparative in other cases.

May be due to these different methodologies, there are differences between the three language classifications. Campbell [23], recognizes similarities between the Andean and Maipurean (Arawakan in the above-mentioned classification), grouping them in a stock named Quechumaran. He also noticed resemblances between the Tupí and Macro-Jê languages, while also proposing a third group, which would be that composed by the Chibchan-Paezan languages. The deeper relationship between these three groups is not resolved.

Greenberg [22] clustered the Tupí together with the Arawakan in a group called Equatorial-Tucanoan. He did not clarify the relationship between this group and the remaining three, but assembled those in a large group called Amerindian, including all the native languages spoken in South and Central America, and a few from North America.

Loukotka's [20] classification agrees with Greenberg's [22] in relation to the close relationship between the Tupí and Arawakan. However, Loukotka groups the Chibchan-Paezan with the Andean languages. The relationship of these two groups and their connections with the Macro-Jê are not detailed. Table S1 (Supporting Information) provides a more detailed classification of the languages belonging to each of these groups according to these and additional authors.

In 2007 a close collaborator of Greenberg, Merritt Ruhlen, published a posthumous revision of his Amerindian linguistic family classification [26]. This work considered all the previous criticisms from other scholars and also new studies, making this new classification somewhat closer to Loukotka's proposition. Given this proximity, the present work will not make use of this more recent study, although it can be seen in comparison to the others in Table S1.

### Genetic markers

Starting from the 678 autosomal microsatellite loci (STRs) reported in [10], 297 were removed from the analyses due to a high (>5%) percentage of missing data for at least one of the populations studied here. The remaining 381 STRs were formatted for the genetic analyses software employed here by using the PGDSpider [27] and in-house written scripts (STR IDs are listed in Table S2).

### Populations and samples

From an initial set of 30 populations studied in [10], five were selected to represent the above-mentioned major linguistic groups as follows: Aymara (2n = 18; Andean), Piapoco (2n = 13; Arawakan), Kogi (2n = 17; Chibchan-Paezan), Kaingang (2n = 7; Jean), Guarani (2n = 10; Tupí). See Table S1 for a detailed classification of these languages and [10] for alternative language names and geographic coordinates of each population.

The selection of a single population to represent a whole linguistic group was based on two assumptions. First, the discrepancies between the three linguistic classifications were observed only at deep branches (involving the final relationship among the five language groups); and second, this procedure reduces the number of parameters of the complex demographic models used here, what is important for both statistical and computational reasons [19].

Ethical approval for the original study from which the STR information was obtained was given in Brazil (Kaingang, Guarani) by the Brazilian National Ethics Commission (CONEP Resolution no. 123/98); in Colombia (Piapoco, Kogi) by the Ethics Commission of Universidad de Antioquia, Medellin, Colombia; and in Chile (Aymara) by the Ethic Commission of Universidad de Chile, Santiago, Chile. Individual and tribal informed oral consent was obtained from all participants, since they were illiterate, and they were obtained according to the Helsinki Declaration. The ethics committees approved the oral consent procedure, as well as the use of these samples in population and evolutionary studies.

### Overview of demographic and genetic modeling

**Figure 1:**
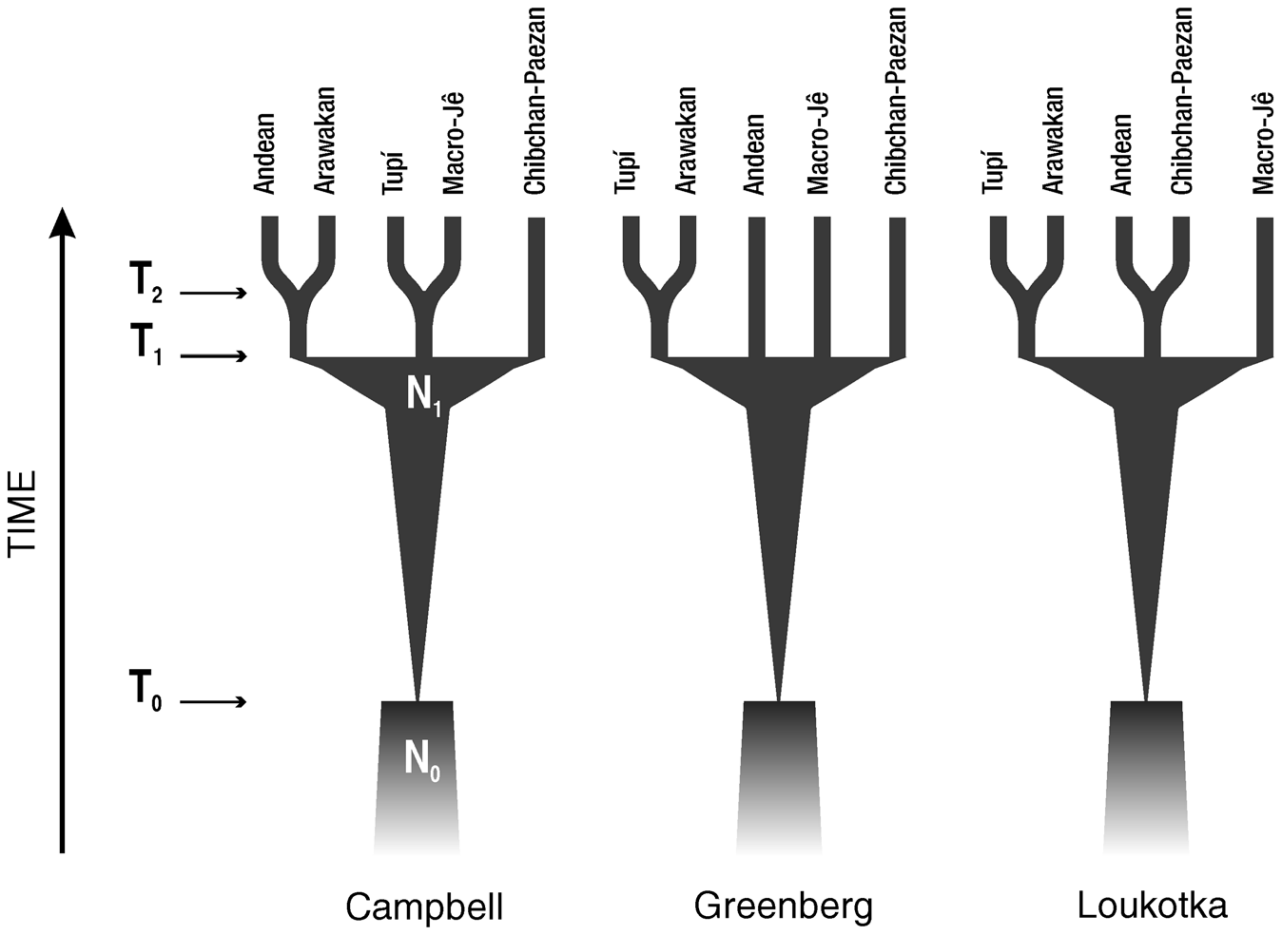
Alternative demographic models tested against the genetic variation in 381 autosomal STRs. Parameters are explained in Table 1. Current average deme size (N_P_) and gene flow (m) between populations are not shown.

Three demographic scenarios (Figure 1) were modeled with Fastsimcoal 1.1.2 [28], which is a simulator of genetic diversity based on the Coalescent [17]. All scenarios presented the same configuration between times T_0_ and T_1_: a small ancestral population of effective size N_0_ (at T_0_) undergoes exponential growth until it reaches effective size N_1_ (at T_1_), time in which the ancestral population undergoes subdivision for the first time as depicted in Figure 1. Further structure arises at T_2_ separating populations that diverged more recently. For each pair of populations in such fission events, an independent T_2_ value was sampled from the prior distribution in each simulation, with a restriction, no sampled value for the date of a more recent fission event (T_2_) could represent older dates than T_1_. Symmetric gene flow was allowed to happen among any pair of populations at a rate of *m*, that is the probability of a gene in the source population to be sent to the sink population. As for T_2_, *m* may also assume different values for each pair of populations. Current average deme size was represented by N_P_, which was assumed to be Gamma (10, 10/N_P_) distributed. The populations were thus allowed to have different sizes and different susceptibility to genetic drift. Time was measured in years, with a generation time of 25 years. Effective population sizes are given in number of diploid individuals. Prior distributions (based on results from recent Native American evolutionary studies) for the main model parameters are given in Table 1.

**Table 1:** Prior distributions of selected model parameters.

Parameter^1^	Distribution	Range	References
T_0_ – Time for the onset of expansion	Uniform	10,000–19,000	[29], [30]
T_1_ –Time for the first emergence of structure	Uniform	800–6,400	[23], [31]
T_2_ –Time for the second emergence of structure	Uniform	800–6,400	[23], [31]
N_0_ – Ancestral effective population size	Uniform	2–1,000	[29]
N_1_ – Effective population (continental) size	Uniform	1,000–100,000	[29]
N_P_ – Current effective deme size	Gamma (10, 10/N_P_)	50–1,000	[29]
m- symmetric migration rate	Uniform	0.00001–0.001.	[29]

1 Time is given in years before present and effective population size in number of diploid individuals (2n). T_1_ and T_2_ prior distributions may present deviations from uniformity, since T_1_>T_2_.

Under a strict stepwise mutation model (SMM), the average STR mutation rate (υ) was set to 6.4×10^−4^ per generation [32]. Since the observed variance between different loci may affect population genetic statistics, and to take this point into consideration, mutation rates were allowed to vary according to the Gamma distribution (α,α/υ; where α is a hyperparameter drawn from an uniform 1–20 distribution). Thus υ was allowed to vary in each simulation and among loci by several orders of magnitude, depending on sampled α values.

### Model choice

The first approach to compare the scenarios was to see if they could generate simulated populations that closely matched the observed data in relation to the distribution of the genetic diversity observed in the 381 loci sampled. The posterior probability of each modeled scenario was then calculated under the ABC framework [18], [19] using the ABCtoolbox [33]. Briefly, for each scenario, 100,000 simulations were generated with Fastsimcoal using the empirical sampling configuration and the previously described models. For each simulation a certain value for each model parameter was sampled from the prior distribution (Table 1) using Fastsimcoal for simulating genetic diversity. Pairwise and global R_ST_, a F_ST_ analogue for STR data which takes into account the difference between STR allelic sizes, were then calculated for each simulated sample and for the empirical dataset with the Arlequin 3.5.1.2 command line version [34] yielding a total 11 summary-statistics. This procedure was conducted with the ABCsampler software implemented with the ABCtoolbox.

The reference tables containing the model parameters used to generate the 100,000 simulations under each scenario and corresponding summary-statistics were then compared to the empirical dataset with the ABCestimator software, also implemented with the ABCtoolbox. This software compares the vectors defined by the summary-statistics estimated for each simulated data set (S) with that estimated for the empirical data (S*) by calculating Euclidian distances δ = ||S-S*|| between them. Half a percent (0.5%) of the simulations matching closest the empirical data were retained for the estimation of the marginal densities of each model. These are then used for the assessment of the posterior odds (Bayes factors; [35]) for each model given the observed data.

To check for potential biases in model choice, 100 additional simulations were generated under each scenario and used as pseudo-empirical data. The same procedure was performed for the empirical data for each of these 300 simulations and the rate of false model inference could then be calculated.

An additional methodology for inferring model posterior probabilities is that proposed by Pritchard et al. [36], which could be described as follows: From the initial 100,000 simulations conducted according to each model, the 100 with smallest associated Euclidian distances to the empirical dataset were retained. This set of 300 simulations was then ranked by ascending Euclidian distances and the posterior probability of a given model was then computed as the proportion of simulations performed under this model included among the 100 first simulations.

### Model parameter estimates

The posterior distributions of the selected parameters (T_0_, T_1_, T_2_, N_0_, N_1_, and N_P_) of the model with higher posterior odds were inferred according to the same framework used for model choice, but with a new reference table with 500,000 simulations. The ABCestimator [33] computes point estimates (mode and median) and confidence intervals (highest posterior density interval) for these distributions. It also checks for potential bias using, in our case, 1,000 pseudo-empirical data, generating a quantiles distribution of the known parameter values in relation to the inferred posterior confidence interval [33], which is then examined statistically for its uniformity according to a Kolmogorov-Smirnov test with α = 0.05 using R [37]. Visual histogram examination was also performed. R was also used to calculate the parameter regression against the summary-statistics, which indicates the proportion of the parameter variance explained by it [38].

## Results

The empirical distribution for the 11 summary-statistics – namely pairwise and global R_ST_s – estimated using the genetic variation of the 381 STRs in the above-mentioned Amerindian populations could be reproduced in the bulk of simulations generated, with no particular better performance for any model. The inference is that all modeled scenarios were able to capture the reality of the STR genome-wide diversity.

Table 2 describes the posterior odds of each scenario according to the two adopted methods to infer posterior probabilities [35], [36]. Both indicate a higher posterior probability for Greenberg's model, followed by Campbell's. Loukotka's model presented virtually no correspondence with the tested genome-wide diversity.

**Table 2:** Posterior probability of three linguistic classifications for South American languages given the genetic diversity of 381 autosomal STRs.

Linguistic classification	Posterior probability (%)	
	Method I [35]	Method II [36]
Campbell [23]	40.3	43.0
Greenberg [22]	59.1	51.0
Loukotka [20]	00.6	6.0

To control for the quality of the model inference, we used the reference table containing the 300 simulations, each 100 generated under a specific model. The known correct model was properly inferred 86% of the times among all inferences performed with the pseudo-empirical data, a rate much higher than that expected by chance (∼33%); the conclusion is that the model fitting procedure was strongly reliable.

**Figure 2:**
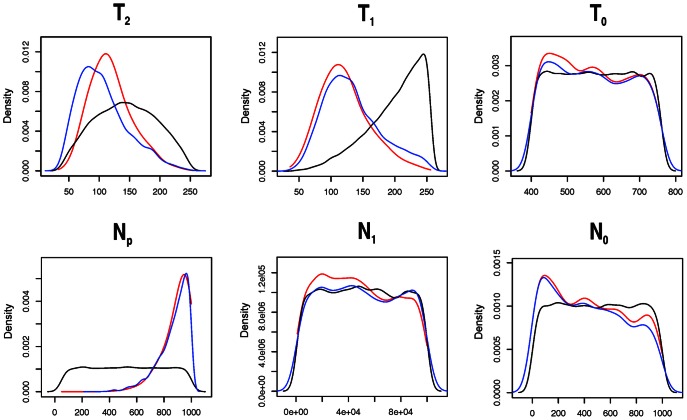
Prior (black), posterior (red) and retained (blue) simulations distributions of time (in generations) and size (2n) of parameters of the demographic model based on Greenberg's [22] language classification.

Figure 2 presents the prior (with all 500,000 runs), retained (0.5%) best simulations and posterior distributions for the selected parameters (T_0_, T_1_, T_2_, N_0_, N_1_, and N_P_) of the demographic model based on Greenberg's language classification. Their characteristics (point estimates and confidence intervals) are given in Table 3 together with the indicators of estimation accuracy. Root mean squared errors (Table 3) indicate that the median was more accurate than the mode in all measures.

**Table 3:** Posterior characteristics of the parameters of the model designed based on Greenberg's [22] classification given the genetic diversity of 381 autosomal STRs.

Parameter	Posterior distribution			Estimation accuracy			
				R^2 2^	RMSE 3		P-value 4
	Mode	Median	HPDI^1^ (95%)		Mode	Median	
T_0_	10,905	14,040	10,136–18,683	0.00	3,625	2,675	0.00
T_1_	2,779	3,094	1,480–5,294	0.03	1,300	1,000	0.05
T_2_	2,666	2,812	800–4,382	0.40	925	850	0. 71
N_0_	52	419	2–985	0.00	423	292	0.47
N_1_	19,905	45,852	2,492–96,020	0.00	40,407	28,474	0.92
N_P_	967	912	709–1,000	0.74	117	106	0.57

1 Highest posterior density interval, which is the continuous interval of parameter values with highest posterior density.

2 Coefficient of determination (R^2^) obtained when regressing the parameter against the summary-statistics.

3 Root mean squared error.

4 P-value considering Kolmogorov-Smirnoff's test for uniformity of posterior quantiles.

**Figure 3:**
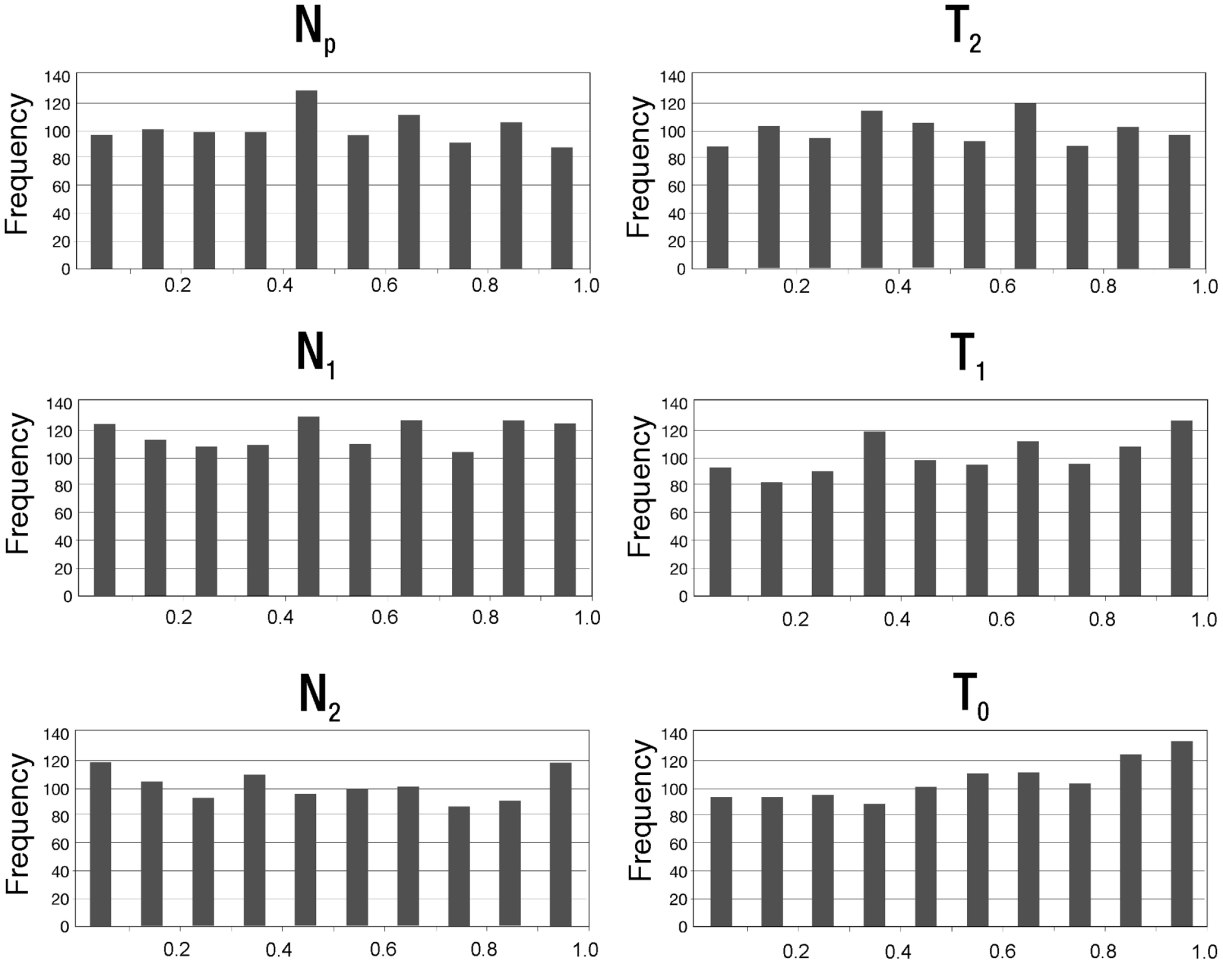
Quantile distributions (x-axis) of the known parameter values as inferred from the posterior distributions for 1,000 pseudo-observed data sets generated under Greenberg's [22] model.

Figure 3 shows the histograms of the posterior quantiles of the model parameters. T_1_, T_2_, and N_P_ present sharp distributions (Figure 2), ideal for ABC estimation. Most of the parameters also present uniform posterior quantiles distribution in the pseudo-empirical dataset (Figure 3) and corresponding Kolmogorov-Smirnov non-significant *p*-values (Table 3). T_2_ and N_P_ also show high R^2^ values (Table 3) suggesting their estimate may be very reliable. In spite of that R^2^ for T_1_ was low. To further test the reliability of the T_1_ estimate, we evaluated the effect of including four additional summary-statistics in its estimation, namely mean and standard deviation of both heterozygosity (H) and number of alleles per locus (K). After this procedure, R^2^ presented a higher value (0.16) and its posterior distribution gave a narrower high posterior density interval (HPDI = 2,835–5,571 years before present-YBP) mostly overlapping with the previous estimate (Table 3). To standardize the analyses performed for parameters' estimation, we will consider only the first estimate for T_1_ and will use the second one just in this step for assuring quality.

The remaining parameter posterior point estimates (T_0_, N_0_, and N_1_) are likely not reliable, since these parameters are poorly explained by the summary statistics (R^2^<10%) (see [38]). The posterior distributions of these parameters did not present clear peaks (Figure 2) and almost no difference from the prior distributions (Tables 1 and 3). However, they present no bias according to the posterior quantiles distribution (Figure 3), except for T_0_, which showed a significant *p*-value for the Kolmogorov-Smirnov test (Table 3).

## Discussion

Campbell's [23], Greenberg's [22], and Loukotka's [20] classifications present marked differences on the relationships of the five South American major linguistic groups. Studies have been conducted to assess which of these propositions presented better correlation with the population relationships suggested by the genetic data. Campbell's and Greenberg's had received genetic support previously ([39] and [40]; [7] and [12], respectively), while Loukotka's classification has not received any. Our results agree with these previous results, since Loukotka's is significantly rejected by the genetic variation observed in a large dataset of fast-evolving autosomal markers widespread along the human genome, while Greenberg's classification receives the greatest support although it is just slightly more adequate than Campbell's (Table 2). The difference between the Loukotkás and the Greenberg's models that may explain why the former is significantly worst fitted to the data is probably the grouping of Andean with Chibchan-Paezan languages.

Comparisons between linguistic and genetic models are very informative for the understanding of human evolution, and may contribute to the knowledge of language evolutionary dynamics; but it should be remembered that they start from quite different methodological assumptions [2]. The main Native American linguistic varieties are classified in well-established language families, but the connection among them to establish major lineages remain controversial. Greenberg's linguistic classification [22] and its multilateral or mass comparison approach have been harshly criticized from a methodological point of view [41]–[43]. According to Greenberg [22], with the exception of the Na-Dene and Eskimo-Aleut language groups, all other Native American languages belong to the single macro-family, named Amerind. This classification was regarded as reductionist by some scholars [44]. In this context, an important issue to consider is the pace of change; language, like other cultural traits, can change in a single generation [5]. The reconstruction of remote language families could be very different if the time period considered is 10,000 or 200,000 YBP [45]. Apart from these caveats and criticisms, it is noteworthy that Reich et al. [46] using information from∼365,000 SNPs genotyped in individuals from 69 Siberian and Native American populations, suggested that the latter descend from at least three streams of Asian gene flow, a compatible scenario with the three major linguistic divisions originally proposed by Greenberg (Amerind, Eskimo–Aleut and Na-Dene).

Greenberg's classification links the Tupí and Arawakan in the Equatorial-Tucanoan group and denies any closer relationship between the Tupí and the Macro-Jê or Arawakan and Andean, as proposed Campbell [23]; or between the Chibchan-Paezan and the Andean, as suggested Loukotka [20].

Notice that for the first time a study relating genetics and language in South America employed the ABC, a statistical framework that allows the use of realistic models which include gene flow and variances in effective population sizes along time and among populations, as well as the use of methods for controlling the quality of the estimates. Therefore, the relationship between any pair of population groups more likely reflects common origin rather than recent gene flow.

As explained in the results, the posterior estimates of T_0_, N_0_, and N_1_ in the model based on Greenberg's classification were not very informative given their confidence intervals being very similar to the prior distributions (Fig. 2 and Table 3) and also not very reliable given their very low coefficients of determination (R^2^). However, since the focus of this investigation was to unravel between-population relationships, these parameters are not of interest and could be considered ‘nuisance parameters’ (see [19]), i.e. they are not of immediate interest but must be accounted for in the analysis of the other parameters.

On the other hand, T_2_, N_P_ and possibly T_1_ estimates from Greenberg's scenario seem to be reliable based on the R^2^ values (Table 3). The current effective deme size (N_P_, 709 to 1,000 diploid individuals) matches Ray's et al. [29] estimates, which range from 751 to 904. T_1_ and T_2_ are exclusive to our models, and it is not possible to compare them with other genetic estimates. The Tupí and Arawakan divergence (T_2_) was estimated to have happened from 800 to 4,382 years ago, with a higher probability of having occurred 2,812 years before present, while the time for the first emergence of structure in South Amerindian groups (T1), indicative of a most recent common ancestor, was dated from 1,480 to 5,294 YBP, with a higher probability at 3,094 years ago (Table 3).

How do these values compare with those obtained from linguistic information? Quechua, an Andean language, emerged 1,150 years before present according to Campbell [23]. The Arawakan group appears to have been formed at 3,000 [24] to 4,000 [47] years ago. The origin of the Chibchan-Paezan languages is dated at sometime between 3,000 and 5,600 before present [23]. Swadesh [48] and Brown [31] estimates for the Chibchan languages emergence are included in this range (5,000 and 4,484 respectively). Jê languages origin is dated between 3,000 to 6,856 years before present according to different authors [24], [48], [49]; more specifically the Kaingang might have emerged 3,000 years ago [24]. The origin of the Tupi-Guarani is dated at some point between 2,000 and 5,000 YBP [24], while Guarani, according to Noelli [50] is 2,000 years old.

Confidence intervals in our genomic approach are large, and those calculated using linguistic data have not been obtained through rigorous statistical criteria. All in all, however, the numbers are not very different, pointing to a relative concordance between the interpopulation genomic and linguistic splits.

## Conclusion

The questions raised in the introduction can now be answered. (a) Greenberg's language classification [22] presents a better fit to the current genome-wide diversity in South America when compared to those of the other linguists, although Campbell's is also compatible with the genomic data; (b) We estimated the time for the emergence of the structure between present day major language groups in South America around 3,100 ago, while the Tupí and Arawakan languages fission seem to have been more recent, around 2,800 years ago; and (c) Although confidence intervals are large, there is general agreement between split times estimated through genomic and linguistic data.

## Supporting Information

Table S1 Classification of the five languages considered in this study. When available, the date of origin of the language is given in parenthesis.
(DOCX)

Table S2 Identification numbers of the 381 STR used in our analyses.
(DOCX)
