# Supplementary material for: A Bayesian Approach to Genome/Linguistic Relationships in Native South Americans
Source: PLoS One. 2013 May 16;8(5):e64099. doi: 10.1371/journal.pone.0064099 (PMC3656118; doi:10.1371/journal.pone.0064099)
Supplement: Table S1 — Classification of the five languages considered in this study. When available, the date of origin of the language is given in parenthesis. (DOCX) [file pone.0064099.s001.docx]

**Supplementary Table S1**. Classification of the five languages considered in this study. When available, the date of origin of the language is given in parenthesis.

| Population | Hierarchic level | Campbell [23] | Urban [24] | Greenberg [22] | Greenberg and Ruhlen [26] | Lewis [25] | Loukotka [20] | Rodrigues [21] |
| --- | --- | --- | --- | --- | --- | --- | --- | --- |
| Kogi | 0 | Chibchan-Paezan (Chibchan + Chocoan) |  |  |  |  |  |  |
|  | 1 | Chibchan (56 centuries ago or sometime after 3000 B.C.) |  | Amerind | Amerind | Chibchan | Languages of Andean tribes |  |
|  | 2 | Chibchan B |  | Chibchan-Paezan | Southern | Aruak | Northern division |  |
|  | 3 | Eastern Chibchan |  | Chibchan | Andean-Chibchan-Paezan | Kogi | Chibcha, stock |  |
|  | 4 | Colombian subgroup |  | Nuclear Chibchan | “Chibchan-Paezan” |  | Arhuaco group |  |
|  | 5 | Northern Colombian group |  | Aruak | Chibchan |  | Koghi (Kogi) |  |
|  | 6 | Arhuacan |  | Kagaba (Kogi) | Nuclear Chibchan |  |  |  |
|  | 7 | Cágaba (Kogi) |  |  | Aruak |  |  |  |
|  | 8 |  |  |  | Kagaba (Kogi) |  |  |  |
| Aymara | 0 | Quechumaran stock (Quechumaran + Aymaran) |  |  |  |  |  |  |
|  | 1 | Aymaran |  | Amerind | Amerind | Aymaran | Languages of Andean tribes |  |

**Supplementary Table S1**. (Cont. 1)

| Population | Hierarchic level | Campbell [23] | Urban [24] | Greenberg [22] | Greenberg and Ruhlen [26] | Lewis [25] | Loukotka [20] | Rodrigues [21] |
| --- | --- | --- | --- | --- | --- | --- | --- | --- |
|  | 2 | Aymara |  | Andean | Southern | Aymara | South Central division |  |
|  | 3 |  |  | Aymara | Andean-Chibchan-Paezan |  | Aymara, stock |  |
|  | 4 |  |  | Aymara | Andean |  | Aymara |  |
|  | 5 |  |  |  | Aymara |  |  |  |
|  | 6 |  |  |  | Aymara |  |  |  |
| Piapoco | 0 | Quechumaran stock (Quechumaran + Aymaran |  |  |  |  |  |  |
|  | 1 | Maipurean | Arawak | Amerind | Amerind | Arawakan | Languages of tropical forest tribes |  |
|  | 2 | Northern division | Maipure (3000 years ago) | Equatorial-Tucanoan | Southern | Maipuran | North Central division |  |
|  | 3 | Upper Amazon branch | Setentrional | Equatorial | “Equatorial-Tucanoan-Ge-Pano-Carib” | Northern Maipuran | Arawak, stock |  |
|  | 4 | Western Nawiki subbranch | Piapoco | Macro-Arawakan | Equatorial-Tucanoan | Inland | Caquetio group |  |
|  | 5 | Piapoko group |  | Arawakan | Equatorial | Piapoco | Piapoco |  |
|  | 6 | Piapoco |  | Maipuran | Macro-Arawakan |  |  |  |

**Supplementary Table S1**. (Cont. 2)

| Population | Hierarchic level | Campbell [23] | Urban [24] | Greenberg [22] | Greenberg and Ruhlen [26] | Lewis [25] | Loukotka [20] | Rodrigues [21] |
| --- | --- | --- | --- | --- | --- | --- | --- | --- |
|  | 7 |  |  | Piapoco | Arawakan |  |  |  |
|  | 8 |  |  |  | Maipuran |  |  |  |
|  | 9 |  |  |  | Piapoco |  |  |  |
| Guarani | 0 | (Similarities between Tupian and Jean languages)^1^ |  |  |  |  |  |  |
|  | 1 | Tupían stock | Tupi-Karib-Macro-Jê (Before 6000 years ago) | Amerind | Amerind | Tupi | Languages of tropical forest tribes | Tronco Tupí |
|  | 2 | Tupí-Guaraní family | Macro-Tupi (Between 3000 and 5000 years ago) | Equatorial-Tucanoan | Southern | Tupi-Guarani | North Central division | Família Tupí-Guaraní |
|  | 3 | Guaraní group | Tupi-Guarani ( 2000 or 3000 years ago) | Equatorial | “Equatorial-Tucanoan-Ge-Pano-Carib” | Subgroup I | Tupi, stock | Guaraní |
|  | 4 | Guaraní language (área) | Guarani | Kariri-Tupi | Equatorial-Tucanoan | Guaraní (Guarani) | Guarani group | “vários” Guaraní (Guarani) |
|  | 5 | “several” Guarani |  | Tupi | Equatorial |  | Guaraní (Guarani) |  |
|  | 6 |  |  | Guaraní | Kariri-Tupi |  |  |  |
|  | 7 |  |  |  | Tupi |  |  |  |

**Supplementary Table S1**. (Cont. 3)

| Population | Hierarchic level | Campbell [23] | Urban [24] | Greenberg [22] | Greenberg and Ruhlen [26] | Lewis [25] | Loukotka [20] | Rodrigues [21] |
| --- | --- | --- | --- | --- | --- | --- | --- | --- |
|  | 8 |  |  |  | Guaraní |  |  |  |
| Kaingang | 0 | (Similarities between Tupian and Jean languages) ^1^ |  |  |  |  |  |  |
|  | 1 | Jean | Tupi-Karib-Macro-Jê (Before 6000 years ago) | Amerind | Amerind | Macro-Ge | Languages of Paleo-American tribes | Tronco Macro-Jê |
|  | 2 | Southern branch | Macro-Jê (At least 5000 or 6000 years ago) | Ge-Pano-Carib | Southern | Ge-Kaingang | Division of Central Brazil | Família Jê |
|  | 3 | Kaingang | Jê (3000 years ago or more) | Macro-Ge | “Equatorial-Tucanoan-Ge-Pano-Carib” | Kaingang | Kaingán, stock | Kaingáng (Kaingang) |
|  | 4 |  | Kaingang | Ge-Kaingan | Ge-Pano-Carib |  | Kaingán (Kaingang) |  |
|  | 5 |  |  | Kaingan | “Ge-Pano” |  |  |  |
|  | 6 |  |  | Kaingan (Kaingang) | Macro-Ge |  |  |  |
|  | 7 |  |  |  | Ge-Kaingang |  |  |  |
|  | 8 |  |  |  | Kaingang |  |  |  |
|  | 9 |  |  |  | Kaingang |  |  |  |

^1^ Campbell [23] sees similarities between Tupian and Jean languages. In our models, those languages were grouped when Campbell’s classification was considered.
