# Supplementary material for: A Bayesian Approach to Genome/Linguistic Relationships in Native South Americans
Source: PLoS One. 2013 May 16;8(5):e64099. doi: 10.1371/journal.pone.0064099 (PMC3656118; doi:10.1371/journal.pone.0064099)
Supplement: Table S2 — Identification numbers of the 381 STR used in our analyses. (DOCX) [file pone.0064099.s002.docx]

**Supplementary Table S2**. List of the microsatellite (STR) loci used in the analysis.

| Loci | Loci | Loci | Loci |
| --- | --- | --- | --- |
| AAAAC001_9 | AGAT119M_1 | ATAA009_8 | D11S1981 |
| AAAT105ZP_2 | AGAT126_5 | ATAA018P_8 | D11S1998 |
| AAAT111_5 | AGAT128_3 | ATAC026P_14 | D11S4459 |
| AAAT121P_8 | AGAT130_5 | ATAC037P_7 | D11S4463 |
| AAAT134_13 | AGAT132_17 | ATAG042_8 | D11S4464 |
| AAC023_3 | AGAT133_7 | ATAG053P_10 | D12S1064 |
| AAC030_3 | AGAT136M_20 | ATAG078P_5 | D12S1300 |
| AACAT001_13 | AGAT140P_9 | ATAG089P_18 | D12S2070 |
| AAT071_3 | ATA009_1 | ATC033_6 | D12S297 |
| AAT107_16 | ATA063_16 | ATC3D09_3 | D12S372 |
| AAT226_16 | ATA069P_14 | ATC4D07_3 | D12S373 |
| AAT238_1 | ATA103C03P_16 | ATCT018_4 | D12S395 |
| AAT245_17 | ATA16D09_2 | ATCT035_20 | D13S1807 |
| AAT246_4 | ATA18C09P_9 | ATCT050_18 | D13S317 |
| AAT253P_12 | ATA20B07_10 | ATGA020_6 | D13S793 |
| AAT256P_6 | ATA21F01_4 | ATGT006Z_10 | D13S796 |
| AAT261_9 | ATA25D12_11 | ATT023_8 | D13S800 |
| AAT268_11 | ATA27C11_11 | ATTT019M_22 | D13S894 |
| AATA019_8 | ATA29E07M_2 | ATTT030_6 | D13S895 |
| AATA053_15 | ATA2E04_1 | CATA002Z_16 | D14S1426 |
| ACT3F12_13 | ATA31F09M_7 | CTAT016_9 | D14S608 |
| AGAT021_2 | ATA38A05_1 | D10S1208 | D14S617 |
| AGAT030P_5 | ATA42G04P_9 | D10S1222 | D14S742 |
| AGAT049P_7 | ATA44F05P_4 | D10S1230 | D15S642 |
| AGAT060_18 | ATA57D10M_3 | D10S1239 | D15S643 |
| AGAT084_12 | ATA58E08ZP_17 | D10S1426 | D15S659 |
| AGAT099P_5 | ATA65H08P_9 | D10S1432 | D15S816 |
| AGAT110P_13 | ATA73A08M_1 | D10S2327 | D16S2616 |
| AGAT115_8 | ATA73C05P_12 | D10S2470 | D16S2621 |
| AGAT116P_14 | ATA80B10Z_10 | D10S677 | D16S2624 |
| AGAT118_1 | ATA85B10P_3 | D11S1392 | D16S3253 |

**Supplementary Table S2**. (Cont. 1)

| Loci | Loci | Loci | Loci |
| --- | --- | --- | --- |
| D16S539 | D20S480 | D3S3039 | D6S305 |
| D16S769 | D20S482 | D3S3045 | D6S474 |
| D17S1294 | D21S1432 | D3S4523 | D6S942 |
| D17S2180 | D21S1437 | D4S1629 | D7S1799 |
| D17S2196 | D21S1440 | D4S1644 | D7S1802 |
| D18S1370 | D21S2052 | D4S1647 | D7S1808 |
| D18S1371 | D22S683 | D4S2368 | D7S1818 |
| D18S1376 | D22S686 | D4S2397 | D7S1824 |
| D18S535 | D2S1328 | D4S2431 | D7S2204 |
| D18S542 | D2S1352 | D4S2623 | D7S3051 |
| D18S851 | D2S1360 | D4S2632 | D7S3056 |
| D18S858 | D2S1394 | D4S3243 | D7S3061 |
| D18S877 | D2S1399 | D4S3248 | D7S3070 |
| D19S246 | D2S1400 | D4S3360 | D7S821 |
| D19S254 | D2S1776 | D5S1456 | D8S1048 |
| D19S559 | D2S1780 | D5S1457 | D8S1108 |
| D19S589 | D2S2944 | D5S1462 | D8S1110 |
| D19S714 | D2S2952 | D5S1501 | D8S1113 |
| D1S1589 | D2S2968 | D5S1505 | D8S1136 |
| D1S1594 | D2S2972 | D5S2488 | D8S1477 |
| D1S1596 | D2S410 | D5S2845 | D8S261 |
| D1S1597 | D2S427 | D5S2849 | D8S592 |
| D1S1653 | D2S434 | D5S817 | D9S1120 |
| D1S1677 | D3S1763 | D5S820 | D9S1121 |
| D1S3462 | D3S1764 | D6S1006 | D9S1122 |
| D1S3669 | D3S2387 | D6S1009 | D9S1838 |
| D1S3721 | D3S2398 | D6S1017 | D9S2157 |
| D1S518 | D3S2409 | D6S1277 | D9S2169 |
| D1S551 | D3S2427 | D6S2410 | D9S922 |
| D20S1143 | D3S2432 | D6S2436 | D9S930 |
| D20S164 | D3S2460 | D6S2439 | D9S934 |

**Supplementary Table S2**. (Cont. 2)

| Loci | Loci | Loci | Loci |
| --- | --- | --- | --- |
| D9S938 | GATA156H01M_8 | GATA51F04P_14 | MFD433-AGAT010_3 |
| F13A1-D6S | GATA157H01_18 | GATA5E06P_9 | MFD442-GTTT002_7 |
| GAAT1A5_2 | GATA165A11M_9 | GATA61F04_9 | MFD455-AAT052_9 |
| GATA036_18 | GATA167C12_12 | GATA63B12P_15 | MFD466-TTA001_16 |
| GATA045_14 | GATA169F02_17 | GATA63F01_2 | NA-D10S-2 |
| GATA060_8 | GATA173A03_18 | GATA6F05P_22 | NA-D12S-1 |
| GATA10H07P_17 | GATA174G01_2 | GATA70F12M_2 | NA-D12S-2 |
| GATA126A06M_2 | GATA175H06M_9 | GATA71E06_11 | NA-D13S-1 |
| GATA129D03M_4 | GATA178C11M_3 | GATA72A06_3 | NA-D14S-1 |
| GATA129G03P_6 | GATA193D02_1 | GATA73B08M_11 | NA-D17S-1 |
| GATA12A08P_5 | GATA194A05M_2 | GATA73D05_18 | NA-D18S-1 |
| GATA131D09_3 | GATA194B06P_2 | GATA73D11P_5 | NA-D1S-2 |
| GATA134F03P_10 | GATA194H05Z_1 | GATA7F09_12 | NA-D1S-3 |
| GATA135F02P_1 | GATA196C10P_10 | GATA81E09_20 | NA-D6S-1 |
| GATA136A04_14 | GATA22F01_15 | GATA81F06_10 | NA-D8S-2 |
| GATA137A12M_7 | GATA22H04M_9 | GATA85D10_18 | NA-D9S-1 |
| GATA137B09_13 | GATA23A02_2 | GATA87D11_7 | SCA10_22 |
| GATA138B05_5 | GATA23G09_1 | GATA8H05_2 | TAAA014P_6 |
| GATA139B09P_5 | GATA27Z_9 | GATA90G05P_10 | TAAAA006_4 |
| GATA140E03_16 | GATA29A06M_2 | GATA90G11M_14 | TACA003_10 |
| GATA141B10M_5 | GATA29B11_11 | GATA91D12M_2 | TAGA002M_2 |
| GATA143C02_15 | GATA29C09P_6 | GATA91G06_14 | TAT028P_7 |
| GATA145G10M_7 | GATA2B02Z_1 | GATA91H01_12 | TAT032Z_15 |
| GATA146B10_3 | GATA30A08M_6 | GGAA19H02_12 | TATC012_8 |
| GATA146D07_3 | GATA30B11_4 | GGAA20F08_1 | TATC028_1 |
| GATA148F04P_21 | GATA31B11_17 | GGAA22C05_12 | TATC057_21 |
| GATA148G10P_2 | GATA31H11P_5 | GGAA23C07_1 | TATG002P_7 |
| GATA149B10M_2 | GATA3H11_8 | GGAA30H04_14 | TCAT006ZP_22 |
| GATA152F04M_3 | GATA4E04_7 | GGAT2G03_3 | TCTA015M_22 |
| GATA152F05L_1 | GATA51A07P_5 | GGAT2G06M_12 | TCTA020_9 |
| GATA153F11_15 | GATA51D11P_5 | GGAT3G09M_9 | TCTA023P_14 |

**Supplementary Table S2**. (Cont. 3)

| Loci | Loci | Loci | Loci |
| --- | --- | --- | --- |
| TCTA025_11 | TTA032Z_6 | TTAT027P_15 | TTTA001M_7 |
| TPO-D2S | TTAT023Z_16 | TTCA004P_8 | TTTA040_3 |
| TTA008P_11 |  |  |  |
